# Personality Traits and Entrepreneurial Intentions: Financial Risk-Taking as Mediator

**DOI:** 10.3389/fpsyg.2022.927718

**Published:** 2022-07-28

**Authors:** Anas A. Salameh, Hameeda Akhtar, Rani Gul, Abdullah Bin Omar, Sobia Hanif

**Affiliations:** ^1^Department of Management Information Systems, College of Business Administration, Prince Sattam Bin Abdulaziz University, Al-Kharj, Saudi Arabia; ^2^Department of Business Administration, Faculty of Management Sciences, International Islamic University Islamabad, Islamabad, Pakistan; ^3^Department of Education, University of Malakand, Chakdara, Pakistan; ^4^Department of Business Administration, National College of Business Administration & Economics (NCBA&E), Lahore, Pakistan

**Keywords:** personality traits, entrepreneurial intentions, extraversion, openness to experience (OE), financial risk taking

## Abstract

The interaction between environment and individual personality determines career. Over the past decades, the role of personality traits in explaining entrepreneurship cannot get much attention of researchers. To fill this gap, this study aims to investigate the effect of personality traits (extraversion, openness to experience, conscientiousness, neuroticism, and agreeableness) on the entrepreneurial intentions (EI) along with the mediating role of financial risk taking (FRT). Sample size consists of 500 students of business and management of different universities of Pakistan, out of which 466 useable questionnaires were collected and analyzed. The results of the study are consistent with conventional wisdom as explored by past studies. In line with past studies and proposed hypothesis, we found that both extraversion and openness to experience have a positive association with FRT, whereas neuroticism, conscientiousness, and agreeableness have negative association with FRT. The results also revealed that there is positive association between FRT and EI; however, FRT did not mediate the relationship between agreeableness and EI.

## Introduction

Pakistan is the 10th largest country in the world according to its labor force (Ahmad et al., 2022). Employment and unemployment facts of the country are the most important contribution for policy and planning functions. According to latest statistics, the 2015 unemployment rate of Pakistan is 5.9%. Unemployment is the biggest challenge faced by most of the developing countries including Pakistan (Gul et al., 2022). Due to this problem, younger people are unemployed and they have less job opportunities. In Pakistan, thousands of students are graduating from different institutes every year; but there are no job opportunities for these graduates. When there are no opportunities for fresh graduates, ultimately, students involve in other activities such as crimes, violence, and many other social immoralities (Farrukh et al., 2017).

The government of Pakistan has been directing its attention to remove these social vices from the country by providing job opportunities and including entrepreneurial skills training in the syllabus of the educational institutes, so that youth have employment skills when they graduate from the universities. The education of entrepreneurship focuses on the developing skills, capability, knowledge of entrepreneurial, as well as intentions and attitudes of entrepreneurial which are consistent with the requirement of the economy (Obschonka et al., 2010). When the economic situation of the country is failing to provide job opportunities to the graduates, then education of entrepreneurship is the best option which supports growth and generates job opportunities (Westhead and Solesvik, 2016).

Gartner (1989) defined the term entrepreneurship as “new entry,” which means the establishment of new business according to the choice of an individual to work for his/her own account and risk. A lot of difficulties are faced in implementing environmental strategy plan even when firms try to meet sustainable supply chain management goals (Davis-Sramek et al., 2022). While firms recognize the importance of utilizing suppliers to meet sustainable supply chain management goals, many find environmental strategy difficult to implement.

Llewellyn and Wilson (2003) stated that the term personality traits have been enlightening the responsive action and industrious action of entrepreneurs. These individuals differentiate themselves as a result of their ability to participate, have interaction, allow, foresee, and advocate transformational alternate when there are scarce resources, diverse situations, and uncertainty (Batool et al., 2021).

However, the role of personality traits in describing entrepreneurship has remained under researched. The motive of this study is to examine the impact of personality traits (extraversion, openness to experience, conscientiousness, neuroticism, and agreeableness) on the entrepreneurial intentions (EI) and the mediating role of financial risk taking (FRT). Risk taking is the desire of a man or woman/organization to actively seize, pursue, and follow opportunities in an uncertain atmosphere *via* accepting the risk involved. Gul et al. (2021c) defined this term as in an uncertain environment how individuals make decision.

Entrepreneurial intention is the major antecedent of entrepreneurship (Lee and Wong, 2004; Abu Elanain, 2008). It is the predictable result of a deliberate conduct. Yoon (2004) defined entrepreneurial intention as first step toward the establishment of a trade (Bukhari et al., 2021b). In Holland (1997) presented theory of career choice, which argue that “Careers are determined by an interaction between individual personality and the environment.” According to John Holland’s Theory, careers depend on individual personality.

## Literature Review

### Personality Traits and Entrepreneurial Intentions

Personality traits were studied extensively to assess the influence of traits of individual on EI. Previous researchers have additionally established a positive relationship between personality characteristics and EI (Antoncic et al., 2015; Karabulut, 2016).

#### Extraversion and Entrepreneurial Intentions

Extroversion supports toward the personality in powering the intuition and also making use of the charismatic visualization of the entrepreneur (Caliendo and Kritikos, 2008; Ahmad and Gul, 2021; Gul et al., 2021a). Entrepreneurs are proposed to possess extroversion as they must be inclined and equipped to keep in touch well with stakeholders. Zhao and Seibert (2006) indicated that entrepreneurs who are more extroverts have a tendency to win investor’s help. Extraversion trait is important for potential entrepreneurs in developing external network’s support (Chandler and Jansen, 1992). Costa et al. (1984) observed that extroverted people are more attracted toward entrepreneurship.

***H1:*** Extraversion positively affects entrepreneurial intentions.

#### Openness to Experience and Entrepreneurial Intentions

People who are more openness to experience trait are not anxious about new challenges, and they have high level of creativity (Chang et al., 2016; Ali et al., 2021; Ayub et al., 2021). Zhao and Seibert (2006) also told that entrepreneurs explore revolutionary ideas and utilize creativity to sort out troubles associated with them. Entrepreneurs were determined to be more open as compared to managerial employees (Nordvik and Brovold, 1998; Hsu and Wang, 2018). These individuals have the quality of creativity which is required to entrepreneurship. Openness to experience revealed a major function in the awareness of opportunity (Irfan, 2021).

***H2:*** Openness to experience positively affects entrepreneurial intentions.

#### Neuroticism and Entrepreneurial Intentions

Individuals who score high on neuroticism regularly show mood swings, recklessness, self-cognizance, arrogance, and depression (Costa and McCrae, 1992; Gul et al., 2021d; Irfan and Khar, 2021). The literature indicates that entrepreneurs have a strong belief in their potential to govern consequences in their environments (Simon et al., 2000), a trait associated with low levels of neuroticism (Pittaway and Cope, 2007). Entrepreneurs who are consistently challenged by any form of problematic circumstances regarding management of resources which are scarce in tandem with pressures of enlightening legitimacy within the face of pressures from that of stakeholders are willing to show off pointless measure of optimism and emotional intelligence (Raja et al., 2004; Bukhari et al., 2021a). In addition, individuals high on neuroticism are terrified by the challenge that has the chance of failing.

***H3:*** Neuroticism negatively affects entrepreneurial intentions.

#### Conscientiousness and Entrepreneurial Intentions

Individuals, who are high in conscientious trait, plan and establish their work, set goals, and continue to give excellent performance, are more likely to become entrepreneur as compared to those who are low in conscientious trait (Hogan and Ones, 1997; Thompson, 2002). The conscientious trait pertains to a person’s diligence, conformance with guidelines/processes and the persistent desire to keep high criteria of performance (Yong, 2007; Wang et al., 2016). Conscientious people are industrious, strive for success and are determined with the aid of a strong knowledge of responsibility that encourages their dependability at work (Gul and Khilji, 2021). Conscientious trait has been found to relate to competitive gains of the organization (Ong and Ismail, 2008).

***H4:*** Conscientiousness positively affects entrepreneurial intentions.

#### Agreeableness and Entrepreneurial Intentions

Entrepreneurs tend to illustrate higher degree of competitiveness than do other styles of business owners, reflecting an absence of agreeableness (Brandstätter, 2011). Agreeable people are trusting, cooperative, and polite (Goldberg, 1990). They are typically lenient, following morality and thoughtful. In comparison with this, individuals who are rating less on courteous attribute are doubtful, self-oriented, and controlling. According to Zhao et al. (2010), individuals having agreeable trait are more concerned with occupations which have public connections such as social work and teaching than to become an entrepreneur.

***H5:*** Agreeableness negatively affects entrepreneurial intentions.

### Personality Traits and Financial Risk Taking

Risk-taking propensity is defined as individual’s willingness to take risk (Sitkin and Weingart, 1995). People with different personalities incline to have different investment preferences which are the outcome of differences in their risk-taking propensities toward investing (Gul et al., 2021b). People who are risk averse invest their money in safer bond while people who are risk taker prefer to invest in risky investment.

#### Extraversion and Financial Risk Taking

Lauriola and Levin (2001) defined the extraversion as it relates to the needs for motivation. It is expressed as a need for recognition and pleasure, together with social potential and dominance. Extraversion was characterized with the aid of the need for diverse, innovative, and tricky situations and abilities and the interest to take physical and societal risks for the sake of such involvements (Zuckerman, 1979).

Extroverted people exhibit higher level of FRT (Zhang et al., 2012). Costa et al. (1984) stated that individuals having extroversion characteristics are positively associated with risk taking. Harlow and Brown (1990) said that extrovert persons are more risk averse whereas introverts are less risk averse.

***H5:*** Extroversion positively affects financial risk taking.

#### Openness to Experience and Financial Risk Taking

Openness to experience is considered as a cognitive concept of risk seeking that entails tolerance to uncertainty, change, and innovation (Zhu et al., 2021). Individuals who score less on this trait are traditional, conservative, and predictable and favor familiar routines to new experiences, due to which they are less likely to take risk (Kowert and Hermann, 1997). We propose that individuals having high level of this trait would be more risk takers.

***H6:*** Openness to experience positively affects financial risk taking.

#### Neuroticism and Financial Risk Taking

Neurotic individuals seem to be much nervous. Lauriola and Levin (2001) said that there is negative relationship between neurotic individuals and risk seeking as a result of its relationship with the anxiousness attribute. Risk seekers might also need some flexibilities (Klein and Kunda, 1994), it can be defined as that they are going to have less scores in expressive sympathy, which is one part of emotions stability (Farrukh and Malik, 2022). Anxious people are prone to exhibit negative feelings, similar to anxiousness, despair, and irritation, instead of being expressively strong. Therefore, it is said that more neurotic people having less risk-taking preferences. The linkage of a low rating with regular risk preferences would suggest that emotional stability influences the stability of risk preferences (Costa and McCrae, 1992).

***H7:*** Neuroticism negatively affects financial risk taking.

#### Conscientiousness and Financial Risk Taking

Conscientiousness specifies a desire for achievements under the provisions of rules and regulations, avoiding uncertainty tolerance (Hogan and Ones, 1997). High degree in conscientiousness could be related to consistent evaluations of risk taking and an aversion to taking risk on uncertain outcomes. High level in conscientiousness is frequently related to risk aversion (Zhang et al., 2013; Irfan and Shahid, 2021), and this study proposes a negative relationship between conscientiousness and risk taking.

***H8:*** Conscientiousness negatively affects financial risk taking.

#### Agreeableness and Financial Risk Taking

Agreeableness can forecast risk propensity to the point, for those traits are negatively related to compulsion, assertiveness, and unfavorable qualities (Lauriola and Levin, 2001). Impulsive decision-makers could make uneven and thoughtless choices and take part in risky behaviors, individuals who have low level of impulsiveness could settle upon safer solutions or schemes (Badar and Irfan, 2018; Ali and Zafar, 2021). Alternatively, risk takers mainly need flexibility (Klein and Kunda, 1994), which specifies low levels of agreeableness.

***H9:*** Agreeableness negatively affects financial risk taking.

### Financial Risk Taking and Entrepreneurial Intentions

As find out that entrepreneurship is traditionally associated with risk taking. In line with Mill (1984), who offered the term entrepreneurship in economics; risk bearing is the important element in distinguishing entrepreneurs from managers. A number of empirical study results support this statement that entrepreneurs are risk takers. Meta-evaluation specify that the risk propensity of entrepreneurs is larger than that of managers. Entrepreneurially inclined individuals have expressively higher scores in risk-taking than the individuals who are not inclined to become entrepreneur (Gurel et al., 2010). One of the most important dimensions of EI is risk taking on the stage of the organization that involves organizations taking risks, committing into ventures that employ most of their assets, and coming into high liability with huge sums of loans. However, it has to be noted that risk taking involves calculating it to make sure it is fine as a substitute of just gambling making use of the assets of the organization (Dess and Lumpkin, 2005).

Risk taking can also be well known as a primary entrepreneurial attribute (Martiarena, 2013), but in comparison with individual entrepreneurs, organizational entrepreneurs who take risks share that threat with their businesses as well. The businesses deliver an additional type of help to the entrepreneurs, i.e., the firm will expect the economic risk while supplying operational and administration help if necessary (Luchsinger and Bagby, 1987). Researches similar to Antoncic and Hisrich (2004) described that risk taking is effective for entrepreneurship. Douglas and Fitzsimmons (2013) learned that risk taking is involving entrepreneurial intention.

***H9:*** Financial risk taking positively effect on entrepreneurial intentions.

### Mediating Role of Financial Risk Taking

Personality traits can describe the differences in EI. Risk taking is the outcome of individual personality (Nicholson et al., 2005), which influence people’s intentions of starting new enterprise ventures. The risk-taking model of Sitkin and Pablo (1992) and the framework of both indicate that personality traits are regarded as major dimension predicating risk-taking behaviors.

Defined the risk perspective of EI as, the degree to which individuals differ in their willingness to take on new unfamiliar situations. Koh (1996) asserts that entrepreneurs are prudent managers of risk were associated with certain business behaviors. Studied the risk-taking propensity in uncertain circumstances. Entrepreneurs have interaction in risky behaviors and seem more inclined to take risks (Adarsha et al., 2021).

Proposed that to become an entrepreneur, a person’s risk profile, financial well-being, profession possibilities, family members, and social relations are important. Investment preferences of individuals depend on the personality traits, which results in the differences in their risk tolerance toward investing. Risk averse traders decide to pay money for safer bonds or invest in a less risky business whereas the risk taker people invest in the risky businesses.

Mancuso (1975) Mentioned that people who become entrepreneurs are usually average risk takers; however, he did not provide empirical support for his point of view. Hence, we proposed that FRT mediates the relationship among big five personality traits and EI.

***H10:*** Financial risk taking mediates the relationship between personality traits and entrepreneurial intentions.

## Research Methodology

The present research was cross-sectional study and quantitative in nature. Data were collected using questionnaires through convenience sampling. A structure questionnaire is designed to collect the data about personality traits and EI for further statistical test. The target population of current research was different university students of business and management of Pakistan. Sample size consists of 500 students of business and management of different universities of Pakistan, out of which 466 useable questionnaires were collected and analyzed as supported. Survey method was used for this study in which data gathered by distributing the questionnaire among different university students of business and management of Pakistan. The collected data analyzed using PROCESS Macro by Hayes and Preacher (2013). Various tests were conducted through SPSS such as Cronbach’s alpha, descriptive statistics, correlation, regression, etc. Simple regression and correlation techniques run to test the hypotheses. The correlation shows the relationship between personality traits and EI. Hayes and Preacher (2013) PROCESS Macro used for mediation analysis.

Personality traits was measured using questionnaire of Soane and Chmiel (2005) and Mayfield et al. (2008). FRT was measured using questionnaire of Hung et al. (2012). EI were measured using questionnaire of Liñán and Yi-Wen (2009). This study used a few control variables related to student’s demographic, such as institution name, gender, age, qualification, and area of specialization.

## Results

### Descriptive Statistics

Descriptive statistics of variables are presented in Table 1. The number of observations is 466 for each of independent variable. In case of E, the mean value is 3.68, having standard deviation of 0.71, whereas O has mean value of 3.67, and deviation from the mean is 0.69. N has the mean value of 3.31 and standard deviation of 0.90. C has the mean value of 3.09 and standard deviation of 1.37, whereas A has the mean value of 3.63 and standard deviation of 0.90. Minimum and maximum values for all independent variables are 1.00 to 5.00.

**TABLE 1:** Descriptive statistics.

Variable	N	Mean	*SD*	Min.	Max.
E	466	3.68	0.71	1.00	5.00
O	466	3.67	0.69	1.00	5.00
N	466	3.31	0.90	1.00	5.00
C	466	3.09	1.17	1.00	5.00
A	466	3.63	0.90	1.00	5.00
EI	466	3.68	0.71	1.00	5.00
SC	466	3.26	0.76	1.00	5.00
FRT	466	3.60	0.79	1.00	5.00
Inst. Name	466		1.39	1.00	5.00
Gender	466		0.30	1.00	2.00
Age	466		0.38	0.00	1.00
Edu.	466		0.48	1.00	2.00
AOS	466		1.48	1.00	5.00

*N, sample size; SD, standard deviation; Min, minimum; Max, maximum; E, extraversion; O, openness to experience; N, neuroticism; C, conscientiousness; A, agreeableness; EI, entrepreneurial intentions; SC, social capital; FRT, financial risk taking.*

### Demographic Analysis

In this study, 500 questionnaires were distributed out of which 466 were returned from different university students of Pakistan, and response rate was 93%. About 59% questionnaire were collected from the students of IIUI, 13, 9, and 10% from students of NUML, Quaid e Azam, CUST, and Other Universities of Pakistan, respectively. The response rate of male students was 30% and female students were 70%. In addition, 18% of the students have age of 20 and below and 82% was 20 and above. The educational level of students was 65% for masters and above and 35% was for bachelor’s degree in which 35% students having specialize in finance, 21% in HRM, 19% in marketing, 7% in IT, and 18% in others.

### Reliability Statistics

This study used Cronbach’s alpha coefficients to test the validity of each variable. The overall reliability of the variables is 0.78, which shows that are variables are reliable. Cronbach’s alpha value of EI is recorded as 0.92, among all the constructs, this value was observed as the highest.

The value of Cronbach’s alpha for mediator variable is 0.57, which is lesser than the acceptable threshold of 0.70. By deleting 1 item of FRT, i.e., FRT33, its alpha value becomes 0.71. In case of independent variables, the overall alpha value of big five personality traits is 0.76. Individual Cronbach’s alpha values of personality traits are as follows: extraversion (0.75), openness to experience (0.78), neuroticism (0.77), conscientiousness (0.80), and agreeableness (0.83).

## ANOVA

Analysis of variance (ANOVA) is used to determine which of our variables are significantly associated with demographic factors so that dummies can be created to control them accordingly. After applying the test, it has been found that our two demographic factors are significant, i.e., institution name and area of specialization for dependent and mediator variables. So, after creating four dummies of these two variables, we control them.

### Correlation Analysis

Table 2 shows the findings of the correlation analysis of the variables which were studied in the current model. The results of variables involved in hypotheses from 1 to 5; impact of independent variable on dependent variable. Personality traits and EI have been observed that the personality traits have shown positive/or negative correlation with EI. Extraversion (E) and openness to experience (O) have been positively correlated with EI having significant values of correlation coefficients of (*r* = 0.163, *p* < 0.01) and (*r* = 0.251, *p* < 0.01), respectively. Neuroticism (N) has shown negative but significant correlation with EI (*r* = 0.279, *p* < 0.01). Conscientiousness (C) has been positively and significantly correlated with EI (*r* = 0.113, *p* < 0.05). The fifth personality trait, i.e., agreeableness (A), has also shown negative but significant correlation with EI (*r* = −0.121, *p* < 0.01).

**TABLE 2:** Correlation analysis.

	In_1	In_3	A-2	A-3	E	O	N	C	A	EI	FRT
In_1	1										
In_3	−0.38**	1									
A_2	0.00	–0.04	1								
A_3	−0.21**	0.37**	−0.24**	1							
E	0.06	–0.06	–0.07	0.11*	1						
O	–0.01	0.0	−0.12**	0.09*	0.35**	1					
N	0.16**	–0.08	–0.03	−0.09*	0.03	0.06	1				
C	0.14**	–0.02	–0.02	–0.02	0.11	0.09*	0.08	1			
A	0.26**	−0.11*	–0.00	−0.13**	0.00	0.03	0.24**	0.17**	1		
EI	−0.11*	0.08	–0.07	0.17**	0.16**	0.25**	−0.27**	0.11*	−0.12**	1	
FRT	0.02	0.11*	−0.12**	0.15**	0.20**	0.32**	−0.18**	–0.05	−0.14**	0.34**	1

N = 466; **Correlation is significant at the 0.01 level (two-tailed). *Correlation is significant at the 0.05 level (Two-tailed).

*Inst. Name, institution name; Edu, education level; AOS, area of specialization; E, extraversion; O, openness to experience; N, neuroticism; C, conscientiousness; A, agreeableness.*

The findings of variables involve in hypothesis from 6 to 10 in which we proposed that impact of independent variables on FRT which was used as a mediator, and it has been detected that extraversion (E) (*r* = 0.200, *p* < 0.01) and O (*r* = 0.322, *p* < 0.01) have positive and significant correlation with FRT. Personality traits, i.e., neuroticism (N), conscientiousness (C), and agreeableness (A), have shown a negative correlation with FRT. Neuroticism (N) and agreeableness (A) have a significant correlation with FRT having values (*r* = −0.183, *p* < 0.01) and (*r* = −0.141, *p* < 0.01), respectively, whereas conscientiousness (C) has weak correlation with FRT (*r* = −0.057, *p* > 0.05). As the variables related to Hypothesis 11 of current study, i.e., the impact of FRT on EI, the finding of correlation analysis shows that FRT has significant and positive association with EI (*r* = 0.348, *p* < 0.01).

The correlation analysis shows that impact of control variables with EI and FRT, it has been found that Inst_1 has negative and significant correlation with EI (*r* = −0.119, *p* < 0.05) and positive but weak correlation with FRT (*r* = 0.028), whereas Inst_3 has positive but weak correlation with EI (*r* = 0.089) and positive and significant correlation with FRT (*r* = 0.111, *p* < 0.05). Area_2 has shown a negative and weak correlation with EI (*r* = −0.07) and negative but significant correlation with FRT (*r* = −0.124, *p* < 0.01), whereas Area_3 has shown a positive and significant correlation with EI (*r* = 0.171, *p* < 0.01) and also positive and significant correlation with FRT (*r* = 0.153, *p* < 0.01).

### Mediation Analysis

The findings for hypothesis (1, 5, and 9) are presented in Table 3, all proposed hypotheses, i.e., 1, 6, 11, and 17, were supported. In line with hypothesis 1, extraversion is positively related to EI (β = 0.13, *t* = 2.11, *p* < 0.05). In hypothesis 6, extraversion is positively related to FRT (β = 0.21, *t* = 4.12, *p* < 0.01). In hypothesis 11, FRT is positively associated with EI (β = 0.40, *t* = 7.19, *p* < 0.01). In line with hypothesis 17, “FRT mediates the positive relationship between extraversion and EI” was found to have an indirect effect on EI *via* extraversion (effect = 0.08). Also, using a Sobel test or normal theory test with a bootstrapped 95% confidence interval (CI), the indirect effect of extraversion on EI was revealed to be significant (Sobel *z* = 3.55, *p* < 0.01) and demonstrated that the bootstrapped CI did not have zero value (0.04, 0.14).

**TABLE 3:** Mediating role of financial risk taking between extraversion and entrepreneurial intentions.

Outcome variable: financial risk taking				β	*se*	*t*	*p*	*R* ^2^
								0.08
Constant				2.72	0.20	13.85	0.000	
Extraversion				0.21	0.05	4.12	0.000	
Institution (IIUI)				0.13	0.08	1.65	0.099	
Institution (CUST)				0.32	0.14	2.31	0.021	
HRM				−0.17	0.09	−1.84	0.067	
Marketing				0.17	0.10	1.65	0.100	
**Outcome variable: entrepreneurial intentions**								
								0.06
Constants				1.68	0.28	5.99	0.000	
Financial Risk Taking				0.40	0.06	7.19	0.000	
Extraversion				0.13	0.06	2.11	0.035	
Institution (IIUI)				−0.25	0.09	−2.63	0.009	
Institution (CUST)				−0.08	0.17	−0.49	0.626	
HRM				−0.00	0.11	−0.04	0.972	
Marketing				0.24	0.12	1.93	0.055	
**Indirect effect of extraversion on entrepreneurial intentions**								
	**Effect**	**Boot SE**		**Boot LLCI**		**Boot ULCI**		
	0.08	0.03		0.04		0.14		
	Normal theory tests for indirect effect							
		**Effect**	***se***	***Z***	***p***			
		0.08	0.02	3.55	0.000			

*Sample size, 466, SE, standard error, LLCI, lower level confidence interval, ULCI, upper level confidence interval, bootstrap sample size, 5000.*

The findings for hypothesis (2, 6, 9, and 10) are presented in Table 4. In line with hypothesis 2, openness to experience is positively related to EI (β = 0.21, *t* = 3.28, *p* < 0.01). In hypothesis 7, openness to experience is positively related to FRT (β = 0.35, *t* = 6.92, *p* < 0.01). In hypothesis 12, FRT is positively associated with EI (β = 0.37, *t* = 6.39, *p* < 0.01. In line with hypothesis 18, “FRT mediates the positive relationship between openness to experience and EI,” was found to have an indirect effect on EI *via* openness to experience (effect = 0.13). Also, using a Sobel test or normal theory test with a bootstrapped 95% CI, the indirect effect of openness to experience on EI was revealed to be significant (Sobel *z* = 4.67, *p* < 0.01) and demonstrated that the bootstrapped CI did not have zero value (0.08, 0.19).

**TABLE 4:** Mediating role of financial risk taking between openness to experience and entrepreneurial intentions.

Outcome variable: financial risk taking				β	*se*	*t*	*p*	*R* ^2^
								0.133
Constant				2.20	0.20	11.13	0.000	
Openness to experience				0.35	0.05	6.92	0.000	
Institution (IIUI)				0.15	0.08	1.90	0.058	
Institution (CUST)				0.27	0.14	2.01	0.045	
HRM				−0.12	0.09	−1.31	0.191	
Marketing				0.19	0.10	1.88	0.060	
**Outcome variable: entrepreneurial intentions**								
								0.060
Constants				1.49	0.27	5.46	0.000	
Financial risk taking				0.37	0.06	6.39	0.000	
Openness to experience				0.21	0.07	3.28	0.001	
Institution (IIUI)				−0.23	0.09	−2.49	0.013	
Institution (CUST)				−0.11	0.17	−0.63	0.528	
HRM				0.02	0.11	0.19	0.850	
Marketing				0.26	0.12	2.11	0.03	
**Indirect effect of openness to experience on entrepreneurial intentions**								
	**Effect**	**Boot SE**		**Boot LLCI**		**Boot ULCI**		
	0.127	0.028		0.078		0.192		
	Normal theory tests for indirect effect							
		**Effect**	***se***	***Z***	***p***			
		0.127	0.027	4.671	0.000			

*Sample size, 466, SE, standard error, LLCI, lower level confidence interval, ULCI, upper level confidence interval, bootstrap sample size, 5000.*

The findings for hypothesis (3, 7, 9, and 10) are presented in Table 5. In line with hypothesis 3, neuroticism is negatively related to EI (β = −0.22, *t* = −4.67, *p* < 0.01). In hypothesis 8, neuroticism is negatively related to FRT (β = −0.17, *t* = −4.12, *p* < 0.01). In hypothesis 13, FRT is positively associated with EI (β = 0.38, *t* = 6.85, *p* < 0.01). In line with hypothesis 19, “FRT mediates the negative relationship between neuroticism and EI” was found to have an indirect effect on EI *via* neuroticism (effect = −0.06). Also, using a Sobel test or normal theory test with a bootstrapped 95% CI, the indirect effect of neuroticism on EI was revealed to be significant (Sobel z = −3.50, *p* < 0.01) and demonstrated that the bootstrapped CI did not have zero value (−0.10, −0.03).

**TABLE 5:** Mediating role of financial risk taking between neuroticism and entrepreneurial intentions.

Financial risk taking				β	se	*t*	*p*	*R* ^2^
								0.076
Constant				4.01	0.15	27.38	0.000	
Neuroticism				−0.17	0.04	−4.12	0.000	
Institution (IIUI)				0.19	0.08	2.41	0.017	
Institution (CUST)				0.27	0.14	1.94	0.053	
HRM				−0.20	0.09	−2.21	0.027	
Marketing				0.20	0.10	1.94	0.054	
**Outcome variable: entrepreneurial intentions**								
								0.186
Constants				2.96	0.28	10.61	0.000	
Financial risk taking				0.38	0.06	6.85	0.000	
Neuroticism				−0.22	0.05	−4.67	0.000	
Institution (IIUI)				−0.17	0.09	−1.85	0.065	
Institution (CUST)				−0.11	0.17	−0.67	0.504	
HRM				−0.05	0.11	−0.42	0.677	
Marketing				0.24	0.12	1.99	0.047	
**Indirect effect of neuroticism on entrepreneurial intentions**								
	**Effect**	**Boot SE**		**Boot LLCI**		**Boot ULCI**		
	−0.062	0.018		−0.103		−0.031		
	Normal theory tests for indirect effect							
		**Effect**	***se***	***Z***	***p***			
		−0.062	0.018	−3.503	0.001			

*Sample size, 466, SE, standard error, LLCI, lower level confidence interval, ULCI, upper level confidence interval, bootstrap sample size, 5000.*

The findings for hypothesis (4, 9, 12, and 15) are presented in Table 6. In line with hypothesis 4, conscientiousness is positively related to EI (β = 0.13, *t* = 3.62, *p* < 0.01). In hypothesis 9, conscientiousness is negatively and insignificantly related to FRT (β = −0.05, *t* = −1.52). In hypothesis 12, FRT is positively associated with EI (β = 0.45, *t* = 8.03, *p* < 0.01). In line with hypothesis 15, “FRT mediates the negative relationship between conscientiousness and EI” was found to have an insignificant indirect effect on EI via conscientiousness (effect = −0.02). Also, using a Sobel test or normal theory test with a bootstrapped 95% CI, the indirect effect of conscientiousness on EI was revealed to be insignificant (Sobel *z* = −1.48) and demonstrated that the bootstrapped CI has zero value (−0.05, 0.00).

**TABLE 6:** Mediating role of financial risk taking between conscientiousness and entrepreneurial intentions.

Outcome variable: financial risk taking				β	*se*	*t*	*p*	*R* ^2^
								0.047
Constant				3.62	0.11	31.61	0.000	
Conscientiousness				−0.05	0.03	−1.52	0.129	
Institution (IIUI)				0.17	0.08	2.04	0.042	
Institution (CUST)				0.28	0.14	1.98	0.048	
HRM				−0.18	0.09	−2.01	0.045	
Marketing				0.23	0.10	2.19	0.029	
**Outcome variable: entrepreneurial intentions**								
								0.171
Constants				1.65	0.24	6.92	0.000	
Financial risk taking				0.44	0.06	8.03	0.000	
Conscientiousness				0.13	0.04	3.62	0.000	
Institution (IIUI)				−0.03	0.10	−3.11	0.002	
Institution (CUST)				−0.15	0.17	−0.89	0.376	
HRM				0.01	0.11	0.04	0.966	
Marketing				0.27	0.12	2.22	0.027	
**Indirect effect of conscientiousness on entrepreneurial intentions**								
	**Effect**	**Boot SE**		**Boot LLCI**		**Boot ULCI**		
	−0.021	0.013		−0.047		0.004		
	Normal theory tests for indirect effect							
		**Effect**	***se***	***Z***	***p***			
		−0.021	0.014	−1.484	0.138			

*Sample size, 466, SE, standard error, LLCI, lower level confidence interval, ULCI, upper level confidence interval, bootstrap sample size, 5000.*

The findings for hypothesis (5, 8, 9, 10) are presented in Table 7. The results show that agreeableness is negatively related to EI (β = −0.04, *t* = −0.79, ns), and thus, hypothesis 5 was not supported. In hypothesis 8, agreeableness is negatively related to FRT (β = −0.13, *t* = −3.19, *p* < 0.01); hence, hypothesis 8 was supported. FRT is positively associated with EI (β = 0.42, *t* = 7.49, *p* < 0.01), and thus, hypothesis was supported. In line with hypothesis 19, “FRT mediates the negative relationship between agreeableness and EI” was found to have an indirect effect on EI *via* agreeableness (effect = −0.06).

**TABLE 7:** Mediating role of financial risk taking between agreeableness and entrepreneurial intentions.

Financial risk taking				β	*se*	*t*	*p*	*R* ^2^
								0.063
Constant				3.93	0.16	24.97	0.000	
Agreeableness				−0.13	0.04	−3.19	0.002	
Institution (IIUI)				0.21	0.08	2.53	0.012	
Institution (CUST)				0.28	0.14	1.97	0.050	
HRM				−0.19	0.09	−2.05	0.041	
Marketing				0.20	0.10	1.95	0.052	
**Outcome variable: entrepreneurial intentions**								
								0.148
Constants				2.23	0.29	7.73	0.000	
Financial Risk Taking				0.42	0.06	7.49	0.000	
Agreeableness				−0.04	0.05	−0.79	0.432	
Institution (IIUI)				−0.22	0.10	−2.28	0.023	
Institution (CUST)				−0.12	0.17	−0.70	0.487	
HRM				−0.01	0.11	−0.11	0.913	
Marketing				0.27	0.12	2.13	0.034	
**Indirect effect of agreeableness on entrepreneurial intentions**								
	**Effect**	**Boot SE**		**Boot LLCI**		**Boot ULCI**		
	−0.055	0.018		−0.092		−0.023		
	Normal theory tests for indirect effect							
		**Effect**	***se***	***Z***	***p***			
		−0.055	0.019	−2.911	0.004			

*Sample size, 466, SE, standard error, LLCI, lower level confidence interval, ULCI, upper level confidence interval, bootstrap sample size, 5000.*

Moreover, using a Sobel test or normal theory test with a bootstrapped 95% CI, the indirect effect of agreeableness on EI was revealed to be significant (Sobel *z* = −2.91, *p* < 0.01) and demonstrated that the bootstrapped CI did not have zero value (−0.09, −0.02), and thus, hypothesis 19 was supported.

## Conclusion and Discussion

### Discussion

This study demonstrated the impact of big five personality traits on EI and mediating role of FRT. Personality traits were divided into five factors in accordance with past research (Brandstätter, 2011; Chia and Liang, 2016). According to the results, personality trait, i.e., extraversion, is positively associated with EI as extraverted people are more sociable and outgoing. The findings of this study are in line with many previous studies (Farrukh et al., 2016).

Conscientiousness is positively associated with EI, whereas openness to experience also has positive association with EI. The findings of these studies are in line with those of previous studies (Zhao et al., 2010; Brandstätter, 2011) and found that people have strong EI who score higher in conscientiousness and openness to experience traits.

The findings of this study are in line with our proposed hypothesis that neuroticism is negatively associated with EI. These findings are opposed to past research (Farrukh et al., 2016) and found that neuroticism did not have impact on EI. The finding of this study did not support our proposed hypothesis that agreeableness is negatively associated with EI, and these findings are in line with previous studies (Farrukh et al., 2016).

The findings of this study revealed that both extraversion and openness to experience have a positive association with FRT, whereas neuroticism, conscientiousness, and agreeableness have negative association with FRT. These findings were consistent with our proposed hypothesis and also consistent with many past researches (Harlow and Brown, 1990; McCrae and Costa, 2008; Pak and Mahmood, 2015).

The results revealed that there is positive association between FRT and EI, and these findings were consistent with the hypothesized model and also consistent with the past studies.

FRT was used as a mediator in this study. FRT mediated the relationship between personality traits, i.e., (extraversion, openness to experience, neuroticism, and conscientiousness) and EI. However, FRT did not mediate the relationship between agreeableness and EI.

### Future Directions

Entrepreneurship is very important for the development and growth of industry such as textile designing and interior designing sectors, and therefore, the current model can be further studied in the future by targeting population of different industry specialization degree programs students of Pakistan or in any other country because in this study, target population was the students of business and administration of different universities of Pakistan.

### Conclusion

This study focuses on the mediating role of FRT between personality traits and EI. Questionnaires were distributed among sample of 500 different university’s students of business and management of Pakistan out of which 466 useable questionnaires were collected and analyzed. Hayes and Preacher (2013) PROCESS Macro was used for correlation and regression analysis. The regression results found that the big five personality traits have a positive and significant impact on EI except agreeableness. However, FRT mediated the association between personality traits and EI.

## Data Availability Statement

The raw data supporting the conclusions of this article will be made available by the authors, without undue reservation.

## Ethics Statement

Ethical review and approval was not required for the study on human participants in accordance with the local legislation and institutional requirements. The patients/participants provided their written informed consent to participate in this study.

## Author Contributions

All authors listed have made a substantial, direct, and intellectual contribution to the work, and approved it for publication.

## Conflict of Interest

The authors declare that the research was conducted in the absence of any commercial or financial relationships that could be construed as a potential conflict of interest.

## Publisher’s Note

All claims expressed in this article are solely those of the authors and do not necessarily represent those of their affiliated organizations, or those of the publisher, the editors and the reviewers. Any product that may be evaluated in this article, or claim that may be made by its manufacturer, is not guaranteed or endorsed by the publisher.

## Appendix

**Table:**

	Big five personality traits					
E1	I really enjoy talking to people.	**1**	**2**	**3**	**4**	**5**
E2	I often feel as if I am bursting with energy.	**1**	**2**	**3**	**4**	**5**
E3	I am a cheerful and high-spirited person.	**1**	**2**	**3**	**4**	**5**
E4	I am a very active person.	**1**	**2**	**3**	**4**	**5**
E5	I make friends easily.	**1**	**2**	**3**	**4**	**5**
O6	I am full of ideas.	**1**	**2**	**3**	**4**	**5**
O7	I have a lot of intellectual curiosity.	**1**	**2**	**3**	**4**	**5**
O8	I carry conversations to a higher level.	**1**	**2**	**3**	**4**	**5**
O9	I often enjoy playing with theories of abstract ideas.	**1**	**2**	**3**	**4**	**5**
N10	Under immense stress and burden, I feel like I am going to pieces.	**1**	**2**	**3**	**4**	**5**
N11	Frequently I feel like I am totally unimportant.	**1**	**2**	**3**	**4**	**5**
N12	Too often, when things go wrong, I get discouraged and feel like giving up.	**1**	**2**	**3**	**4**	**5**
N13	I often feel tense and anxious.	**1**	**2**	**3**	**4**	**5**
C14	I am pretty good about paving myself so as to get things done in time.	**1**	**2**	**3**	**4**	**5**
C15	I keep my belonging neat and clean.	**1**	**2**	**3**	**4**	**5**
C16	I waste lot of time before setting down to work.(R)	**1**	**2**	**3**	**4**	**5**
C17	I am always dependable and organized.	**1**	**2**	**3**	**4**	**5**
A18	I generally try to be thoughtful and considerate.	**1**	**2**	**3**	**4**	**5**
A19	I never get into arguments with my family and co-workers.	**1**	**2**	**3**	**4**	**5**
A20	Some people think of me as cold and calculating.(R)	**1**	**2**	**3**	**4**	**5**
A21	Most people think that I am not selfish and egotistic.	**1**	**2**	**3**	**4**	**5**
	**Financial risk taking**					
FR22	I like to take chances, although I may fail.	**1**	**2**	**3**	**4**	**5**
FR23	Although a new thing has a high promise of reward, I do not want to be the first one who tries it. I would rather wait until it has been tested and proven before I try it.(R)	**1**	**2**	**3**	**4**	**5**
FR24	When I have to make a decision for which the consequence is not clear, I like to go with the safer option although it may yield limited rewards.(R)	**1**	**2**	**3**	**4**	**5**
FR25	I like to try new things, knowing well that some of them will disappoint me.	**1**	**2**	**3**	**4**	**5**
FR26	To earn greater rewards, I am willing to take higher risks.	**1**	**2**	**3**	**4**	**5**
	**Entrepreneurial intentions**					
EI27	I am ready to do anything to be an entrepreneur.	**1**	**2**	**3**	**4**	**5**
EI28	My professional goal is to become an entrepreneur.	**1**	**2**	**3**	**4**	**5**
EI29	I will make every effort to start and run my own firm.	**1**	**2**	**3**	**4**	**5**
EI30	I am determined to create a firm in the future.	**1**	**2**	**3**	**4**	**5**
EI31	I have very seriously thought of starting a firm.	**1**	**2**	**3**	**4**	**5**
EI32	I have the firm intention to start a firm someday.	**1**	**2**	**3**	**4**	**5**
